# A Novel In Vitro Culture Model System to Study Merkel Cell Polyomavirus–Associated MCC Using Three-Dimensional Organotypic Raft Equivalents of Human Skin

**DOI:** 10.3390/v13010138

**Published:** 2021-01-19

**Authors:** Amanda S. W. Loke, B. Jack Longley, Paul F. Lambert, Megan E. Spurgeon

**Affiliations:** 1 McArdle Laboratory for Cancer Research, Department of Oncology, University of Wisconsin School of Medicine & Public Health, Madison, WI 53705, USA; aloke@wisc.edu; 2 Department of Dermatology, University of Wisconsin School of Medicine & Public Health, Madison, WI 53705, USA; bjlongley@dermatology.wisc.edu

**Keywords:** Merkel cell polyomavirus, Merkel cell carcinoma, Merkel cells, DNA tumor virus, human polyomavirus, organotypic rafts, skin equivalents

## Abstract

Merkel cell polyomavirus (MCPyV) is a human polyomavirus causally linked to the development of Merkel cell carcinoma (MCC), an aggressive malignancy that largely arises within the dermis of the skin. In this study, we recapitulate the histopathology of human MCC tumors in vitro using an organotypic (raft) culture system that is traditionally used to recapitulate the dermal and epidermal equivalents of skin in three dimensions (3D). In the optimal culture condition, MCPyV+ MCC cells were embedded in collagen between the epidermal equivalent comprising human keratinocytes and a dermal equivalent containing fibroblasts, resulting in MCC-like lesions arising within the dermal equivalent. The presence and organization of MCC cells within these dermal lesions were characterized through biomarker analyses. Interestingly, co-culture of MCPyV+ MCC together with keratinocytes specifically within the epidermal equivalent of the raft did not reproduce human MCC morphology, nor were any keratinocytes necessary for MCC-like lesions to develop in the dermal equivalent. This 3D tissue culture system provides a novel in vitro platform for studying the role of MCPyV T antigens in MCC oncogenesis, identifying additional factors involved in this process, and for screening potential MCPyV+ MCC therapeutic strategies.

## 1. Introduction

Merkel cell polyomavirus (MCPyV) was discovered in 2008 in the context of a rare but highly lethal form of skin cancer, Merkel cell carcinoma (MCC) [1]. MCC is a cancer with neuroendocrine characteristics that was originally described as “trabecular carcinoma of the skin” [2] but later re-named to its current moniker because it expresses biomarkers also expressed in Merkel cells, a specialized mechanoreceptor cell within the skin involved in touch sensation [3]. MCPyV is one of multiple recently identified human polyomaviruses (reviewed in [4,5]). Similar to other polyomaviruses, MCPyV has a double-stranded, circular DNA genome encoding “early” and “late” genes [1]. MCPyV is ubiquitous in the human population, as indicated by the high frequency of detection of MCPyV DNA genomes from skin swabs and the high percentage of seropositivity [6,7]. Potential cell types that have been proposed to support viral infection include epithelial cells, fibroblasts, pre-B cells, and pro-B cells [8,9,10,11]. Within the skin, studies suggest that the most likely tropism of MCPyV is either epithelial cells and/or dermal fibroblasts [6,11,12]. To date, only dermal fibroblasts [8] and HEK293 cells [13] have been reported to support the viral life cycle of MCPyV in vitro and result in the production of infectious progeny virions capable of serial transmission. The development of in vitro tissue culture conditions that recapitulate the histopathology of MCC could help scientists study the role of MCPyV in the genesis of this deadly disease.

MCPyV is strongly implicated in having an etiological role in the development of MCC, making MCPyV the only polyomavirus currently known to cause human cancers (reviewed in [14]). In at least 80% of MCC tumors, the MCPyV viral genome is found clonally integrated into the host genome (MCPyV+ MCC) [1]. The existence of these clonal events, shared among cells of the same cancer specimen, led investigators to conclude that MCPyV DNA integration must be an early event in the genesis of these cancers. These integration events lead to the loss of expression of the viral late proteins but continued expression of two viral early proteins: small T antigen (ST) and truncated forms of large T antigen (LT) [15,16]. Shuda et al. reported that the different truncations of MCPyV LT found in different MCPyV+ MCC universally result in the loss of the DNA helicase domain, which is essential for viral DNA replication [16]. However, these truncated LT molecules retain the LxCxE motif that mediates binding to the cellular, retinoblastoma (Rb) tumor suppressor protein [16,17]. Binding of MCPyV LT to Rb inhibits Rb’s capacity to regulate the activity of the transcription factor E2F, a property that contributes to the transforming capacity of other polyomaviruses [18]. Multiple investigators have uncovered the transforming/tumorigenic properties of MCPyV ST and/or LT in a variety in vitro and in vivo settings [19,20,21,22,23,24,25,26]. Nevertheless, perhaps the strongest evidence for the role of MCPyV ST and LT in the cancer phenotype of MCPyV+ MCC is the observation that silencing their expression induces cell cycle arrest and apoptosis [27] and causes MCPyV+ MCC cells to undergo phenotypic changes and apparent conversion to a neuron-like cell type [28], demonstrating the critical role of these viral proteins in MCC.

To better understand the involvement of the T antigens in the development of MCPyV+ MCC, a great deal of effort has been focused toward developing in vivo animal models of MCC utilizing transgenic mice [23,25,26]. These in vivo studies provide strong evidence for the tumorigenic potential of ST and truncated LT; however, to date, there are no transgenic mouse lines described in the literature that actually develop MCC. That being said, there is one tantalizing study that demonstrated MCC-like cellular aggregates arising in the skin of newborn transgenic mice with enforced expression of MCPyV ST and the Merkel cell specification factor ATOH1 [26]. Unfortunately, these newborn mice did not survive to give rise to stable lines of transgenic mice. In addition to in vivo murine models, several human tumor-derived MCPyV+ MCC cell lines have been established in vitro and are critical tools in the identification of co-factors and biological pathways that lead to oncogenesis [29,30,31,32]. 

Despite the significant advances in developing tools to study MCPyV+ MCC, the field of MCPyV+ MCC research still lacks an in vitro model system to study the dynamics of MCC biogenesis, cell–cell interactions, and molecular mechanisms in a physiologically relevant, three-dimensional (3D) tissue context. The current paradigm suggests that MCPyV infections of human skin, most likely in epidermal keratinocytes or dermal fibroblasts [8,11,12,13,33], facilitate cutaneous MCC neoplastic progression following the integration of viral genome into the host genome. MCC most frequently arises in the dermis [34,35], and there is evidence that cellular communication between MCPyV+ MCC cells and keratinocytes may play a role in MCPyV T antigen–mediated MCC carcinogenesis [28]. Therefore, an in vitro model that recapitulates the 3D spatial dynamics of human skin might be an ideal tissue culture model in which to study MCPyV+ MCC. 

Organotypic “raft” cultures, also known as skin equivalents, are in vitro 3D cultures that allow one to recapitulate the stratified epithelium and dermal component of the skin in a tissue culture setting [36]. Keratinocytes are seeded onto a dermal equivalent that contains fibroblasts and, upon being raised to the air–liquid interface, reproduce the stratification and terminal differentiation process of human skin. The resulting rafts histologically resemble human skin with a cornified epidermal equivalent arising on top of a dermal equivalent containing human fibroblasts. These raft models are powerful tools that have been used in our lab and others to study the epithelial-tropic human papillomaviruses (HPVs), the life cycles of which are tied to the terminal differentiation process found within the epidermis [37,38,39,40,41,42,43,44]. Rafts have also been used to study herpes simplex virus (HSV) [45,46], adeno-associated virus type 2 [47], alphaherpesvirus [48], and Epstein–Barr virus [49,50,51,52]. 

In this study, we adapted our previously established raft protocol [40,41] to develop a novel in vitro 3D model system for the co-culture of MCPyV+ MCC cell lines. We tested several approaches for incorporating MCPyV+ MCC into rafts, including co-culture of MCC cells with keratinocytes, with fibroblasts, and/or with or without being embedded in collagen. Our studies identified an optimal co-culturing method in which MCPyV+ MCC cultured in organotypic rafts developed lesions within the dermal layer that closely resemble human MCC pathology. This novel three-dimensional tissue culture system provides a new in vitro platform for studying the role of MCPyV T antigens in MCC biogenesis and cellular interactions and crosstalk between MCC cells and other cells present in human skin and testing potential MCC therapeutic agents. 

## 2. Materials and Methods

### 2.1. Cell Lines and Culture Conditions

The Merkel cell polyomavirus–positive Merkel cell carcinoma (MCPyV+ MCC) cell line MKL-1 (catalog number #09111801) was obtained from Sigma-Aldrich (St. Louis, MO, USA). MKL-1 cells were cultured in RPMI 1640 media supplemented with 10% fetal bovine serum (FBS; Hyclone #SH30071.03; GE Healthcare Life Sciences, Marlborough, MA, USA) and 100 μg/mL penicillin/streptomycin. 

The spontaneously immortalized human keratinocyte cell line, NIKS, is derived from normal human neonatal foreskin keratinocytes as described in Allen-Hoffmann et al. [38]. NIKS cells were maintained on mitomycin C–treated J2 3T3 feeder cells in complete F medium (3 parts F12:1 part Dulbecco’s modified Eagle’s medium (DMEM) supplemented with 5% FBS, 24 μg/mL adenine, 8.4 ng/mL cholera toxin, 10 ng/mL epidermal growth factor (EGF), 2.4 μg/mL hydrocortisone, 5 μg/mL insulin, 100 μg/mL penicillin/streptomycin) as described [40,41]. 

Early passage human foreskin fibroblasts (EF-1-F) were obtained from Dr. Lynn Allen-Hoffmann and cultured in F12 media supplemented with 10% FBS and 100 μg/mL penicillin/streptomycin. All cells were maintained in a humidified atmosphere of 5% CO_2_ at 37 °C.

### 2.2. Organotypic Raft Culture 

**Figure 1:**
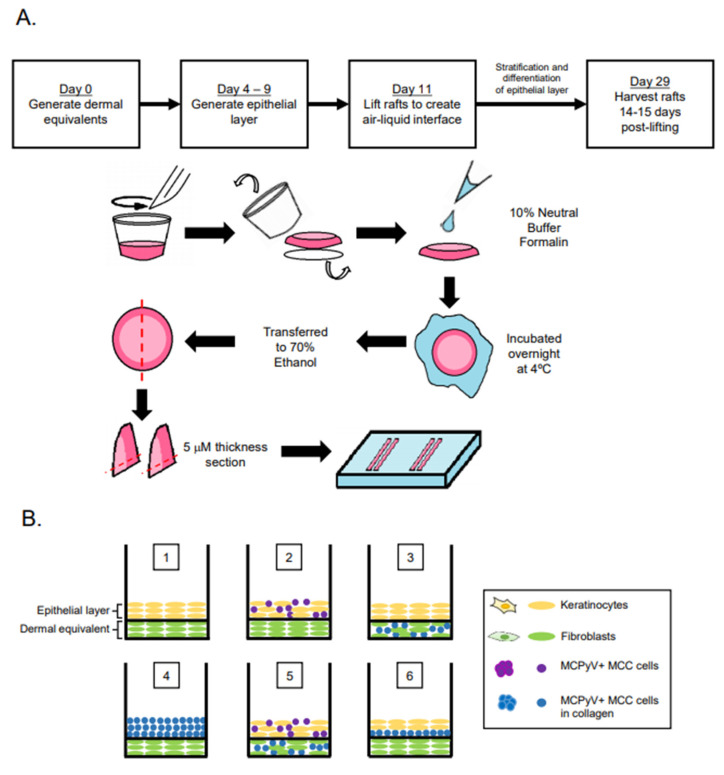

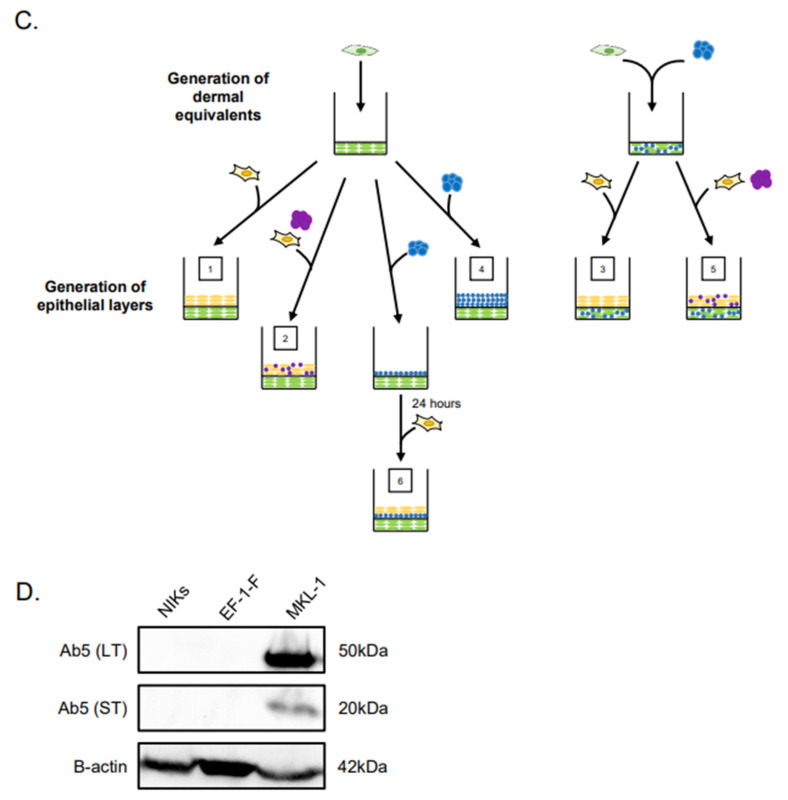
Experimental design of Merkel cell polyomavirus–positive (MCPyV+) Merkel cell carcinoma (MCC) culture in organotypic rafts. (**A**) General workflow and timeline of 3D organotypic raft co-culture with MCPyV+ MCC. Rafts were harvested at the endpoint using the process depicted; paraffin-embedded and 5 µM-thick sections were cut from a paraffin block and laid onto slides for hematoxylin and eosin (H&E) and immunostaining analyses. (**B**) Experimental organization/setups of rafts generated. The six conditions tested are depicted. Cell types included in the various raft setups include NIKS keratinocytes (yellow), early passage human foreskin fibroblasts (EF-1-F) fibroblasts (green), MCPyV+ MCC cells without collagen (purple), and MCPyV+ MCC cells in collagen (blue). (**C**) Experimental workflow for each raft setup depicted in (**B**). Dermal equivalents were generated with either EF-1-F or EF-1-F/MKL-1 mix and left to culture for 4–7 days. The epithelial layer was then generated by layering NIKS epithelial cells or epithelial cells with MKL-1 cells. For Setup 4, MKL-1 cells were suspended in collagen and added to the dermal equivalent. For Setup 6, an intermediate layer of MKL-1 cells in collagen was added and allowed to settle for 24 h, followed by the addition of NIKS keratinocytes. (**D**) Immunoblot analysis for the MCPyV large T antigen (LT) and small T antigen (ST) proteins in cell types used in raft studies. Protein lysates from NIKS keratinocytes, EF-1-F fibroblasts, and the MCPyV+ MCC cell line MKL-1 were analyzed by immunoblotting with the Ab5 mouse monoclonal antibody. An immunoblot for β-actin was included as a loading control.

Organotypic (raft) cultures were generated by adapting our lab’s protocol for studying the HPV life cycle [40,41]. Briefly, dermal equivalents with either 6.1 × 10^4^ EF-1-F fibroblasts or a mixture (1:1 mixture; 3.05 × 10^4^ cells from each cell line) of EF-1-F and MCPyV+ MCC cells suspended in rat tail collagen type I (Millipore Sigma #08-115, Burlington, MA, USA) were generated and plated onto transwells (Corning™ Transwell™ Multiple Well Plate pore size 0.4 μm, Fisher Scientific #07200170, Waltham, MA, USA) with fibroblast medium (F12 supplemented with 10% FBS and pen/strep) for 4–7 days. In Setups 7 and 8, where fibroblast cells were not included in the dermal equivalents, the same collagen mixture was generated without EF-1-F cells. The same volume of collagen was then plated on to the transwells. To generate an intermediate layer with MCPyV+ MCC cells in Setup 6, 1 × 10^5^ MKL-1 cells were suspended in collagen (supplemented with 10% 10× F-12 media, 10% FBS, 100 μg/mL penicillin/streptomycin, and 10 N sodium hydroxide) and added onto the dermal equivalent 24 h before the addition of keratinocytes (1 × 10^5^ NIKS cells). To form the epithelial layer, 2.1 × 10^5^ keratinocytes (NIKS) or 2.1 × 10^5^ MKL-1 MCC cells in collagen were added in keratinocyte plating media (3 parts F12:1 part DMEM supplemented with 0.5% FBS, 24 μg/mL adenine, 8.4 ng/mL cholera toxin, 2.4 μg/mL hydrocortisone, 5 μg/mL insulin, 1.22 mM Ca^2+^, and pen/strep). In Setups 2 and 5, where NIKS and MKL-1 cells were mixed in a 1:1 ratio to form the epithelial cell layer, 1.05 × 10^5^ cells each of NIKS and MKL-1 cells were combined to keep the total cell number (2.1 × 10^5^) used to form the epithelial layer constant across conditions. Four days post generation of the epithelial layer, rafts were lifted and placed on top of sterile cotton pads to raise the raft to the air–liquid interface. Post-lifting, rafts were cultured with cornification medium (3 parts F12:1 part DMEM supplemented with 5% FBS, 24 μg/mL adenine, 8.4 ng/mL cholera toxin, 2.4 μg/mL hydrocortisone, 5 μg/mL insulin, 1.22 mM Ca^2+^, and pen/strep) containing 10 μM C8:0 (1,2-dioctanoyl-sn-glycerol, Santa Cruz sc-202397A, Dallas, TX, USA). Culturing of rafts continued for an additional 14 days. On the day of harvest, cornification media supplemented with 10 μm bromodeoxyuridine (BrdU) (Sigma Aldrich, #B5002, St. Louis, MO, USA) was added to the rafts and incubated for 8 h to label newly synthesized DNA for later detection using immunohistochemistry (Figure 1A).

### 2.3. Immunofluorescence and Immunohistochemistry 

Organotypic rafts tissues were harvested as previously described [41] and as outlined in Figure 1A. Briefly, upon removing rafts from the transwell membrane with a scalpel, rafts were fixed in agar containing 10% neutral buffered formalin and incubated at 4 °C overnight before being transferred to 70% ethanol solution. Samples were then embedded in paraffin and sectioned into 5 µM sections. For immunofluorescence, tissue sections were deparaffinized and antigen retrieval was performed by boiling in 10 mM Tris-EDTA buffer pH 9.0 for 20 min. The sections were then blocked with 5% non-fat milk in PBS and 5% goat serum (Jackson Immunoresearch Laboratories #005-000-121, West Grove, PA, USA). Tissues were then washed and stained overnight at 4 °C with primary antibodies at dilutions described in Section 2.4. Antibodies and Reagents. For immunofluorescence, AlexaFluor secondary antibodies were added and incubated for 1 hour at room temperature. Hoechst dye was used to stain cellular nuclei and slides were mounted in Prolong mounting media (Thermo Fisher Scientific P36970, Waltham, MA, USA). For immunohistochemistry, tissue sections were deparaffinized and antigen retrieval was performed by boiling in 10 mM citrate buffer pH 6.0 for 20 min followed by quenching with 3% hydrogen peroxide in 1× PBS for 15 min. Blocking and immunodetection was done using reagents included in the VECTASTAIN^®^ Elite^®^ ABC Universal Kit Peroxidase (Horse Anti-Mouse/Rabbit IgG) kit (Vector Laboratories PK-6200, Burlingame, CA, USA). Incubation times for both primary and secondary antibodies were the same as those used for immunofluorescent detection. Counterstaining was done using Hematoxylin QS Counterstain (Vector Laboratories H-3404, Burlingame, CA, USA) and slides were mounted in ThermoScientific Cytoseal™ XYL (Thermo Fisher Scientific, # 83124, Waltham, MA, USA). All washes for immunofluorescence and immunohistochemistry were performed with 1× PBS. All slides were imaged with the Zeiss AxioImager M2 microscope using the AxioVision software version 4.8.2 (Jena, Germany).

### 2.4. Antibodies and Reagents

The following antibodies were used for immunofluorescence detection: Keratin 14 (K14, BioLegend #905301, San Diego, CA, USA; 1:1000), Keratin 10 (K10, BioLegend #905401, San Diego, CA, USA; 1:1000), Keratin 8 (K8, Developmental Studies Hybridoma Bank TROMA-1, Iowa City, IA, USA; 1:200), MCPyV large T antigen CM2B4 (Santa Cruz Biotechnology sc-136172, Dallas, TX, USA; 1:500), and smooth muscle actin (Monoclonal anti-actin, α-smooth muscle, MilliporeSigma A2547, Burlington, MA, USA; 1:400). The following antibodies were used for immunohistochemistry detection: bromodeoxyuridine (BrdU, MilliporeSigma #NA61, Burlington, MA, USA; 1:50) and minichromosome maintenance protein 7 (MCM7, Thermo Fisher Scientific #MS862, Waltham, MA, USA; 1:200). Fluorescent detection was done using Alexa Fluor anti-mouse 488 1:1000 (#A11011), anti-rabbit 546 1:1000 (#11010) and 647 1:750 (#A21244), and anti-rat 647 1:750 (#A21247) from Molecular Probes (Eugene, OR, USA). Secondary detection for immunohistochemistry was performed according to the procedure included in the VECTASTAIN^®^ Elite^®^ ABC Universal Kit Peroxidase (Horse Anti-Mouse/Rabbit IgG) kit. Both primary and secondary antibodies were diluted in their respective blocking reagents as detailed in Section 2.3. 

### 2.5. Immunoblotting

Immunoblotting was used to verify MCPyV T antigen expression in cell lines prior to culturing in organotypic rafts. Cells were harvested and lysed with RIPA buffer (150 mM 1 M NaCl, 1& Nonidet P-40, 0.5% sodium deoxycholate, 0.1% SDS, 25 mM Tris pH 7.4 in H_2_O) with 50× protease inhibitor cocktail (PIC) and 100 µM PMSF. Protein concentrations were determined using the Bio-Rad Protein Assay (Bio-Rad, #5000006, Hercules, CA, USA). Protein lysates (40 µg total protein per sample) were resolved on 12% TGX FastCast acrylamide gels (Bio-Rad, #1670175, Hercules, CA, USA) and then transferred to 0.45 µM nitrocellulose membrane (Bio-Rad, #1620145, Hercules, CA, USA). The membrane was blocked with 5% non-fat milk in TBS supplemented with 0.1% Tween-20 (TBS-T). MCPyV large T (LT) and small T (ST) antigen expression was detected with primary antibody Ab5 (generous gift from Dr. James DeCaprio, described in [19]) at 1:5000 and incubated overnight at 4 °C with β-actin antibody (Abcam, ab75186) used as a loading control. To detect the bands on the membrane, anti-mouse (Jackson Immunoresearch #115-036-071) and anti-rabbit (Vector Labs #PI-1000) horseradish-peroxidase-conjugated secondary antibodies were added at 1:10,000 dilution and incubated at room temperature for 1 h. To visualize immune complexes, Clarity Western ECL chemiluminescent substrate (Bio-Rad, #170-5061, Hercules, CA, USA) was applied and the blots visualized using a ChemiDoc Universal Hood III using the ImageLab 6.0.1 program (Bio-Rad, Hercules, CA, USA).

## 3. Results

### 3.1. Experimental Design of MCC Culture in Organotypic Rafts 

Our overall experimental design was to culture MCC cells in raft cultures using our standard laboratory protocol, in which rafts are generated and then raised to the air–liquid interface at the indicated time and cultured in stratification medium until the experimental endpoint (Figure 1A). However, we adapted this protocol in order to culture MCPyV+ MCC cells within different tissue compartments, either in the dermal equivalent or the epithelial equivalent, and in the presence or absence of human keratinocytes (the spontaneously immortalized NIKS cell line) and/or human fibroblasts (EF-1-F cells) using several different raft designs/setups (Figure 1B,C). The MCPyV+ MCC cell line included in rafts was MKL-1, a classical MCC cell line derived from a metastatic MCC tumor isolated from a 26-year-old male [31]. The expression of T antigens in the MCPyV+ MCC cell line, but not in NIKS keratinocytes and EF-1-F fibroblasts, was verified by immunoblotting with an antibody (Ab5) that detects both MCPyV LT and ST. Expression of truncated LT and ST was only observed in the MKL-1, as expected (Figure 1D). At the end of the culture period, replicates (*n* = 2) of rafts generated under each of the setup conditions were harvested for analysis (Figure 1A) and tissue sections were stained with hematoxylin and eosin (H&E) in order to observe histology. 

### 3.2. MCPyV+ MCC-Like Lesions Formed in Dermal Layer of Organotypic Raft Cultures but Not in the Epithelial Layer

**Figure 2:**
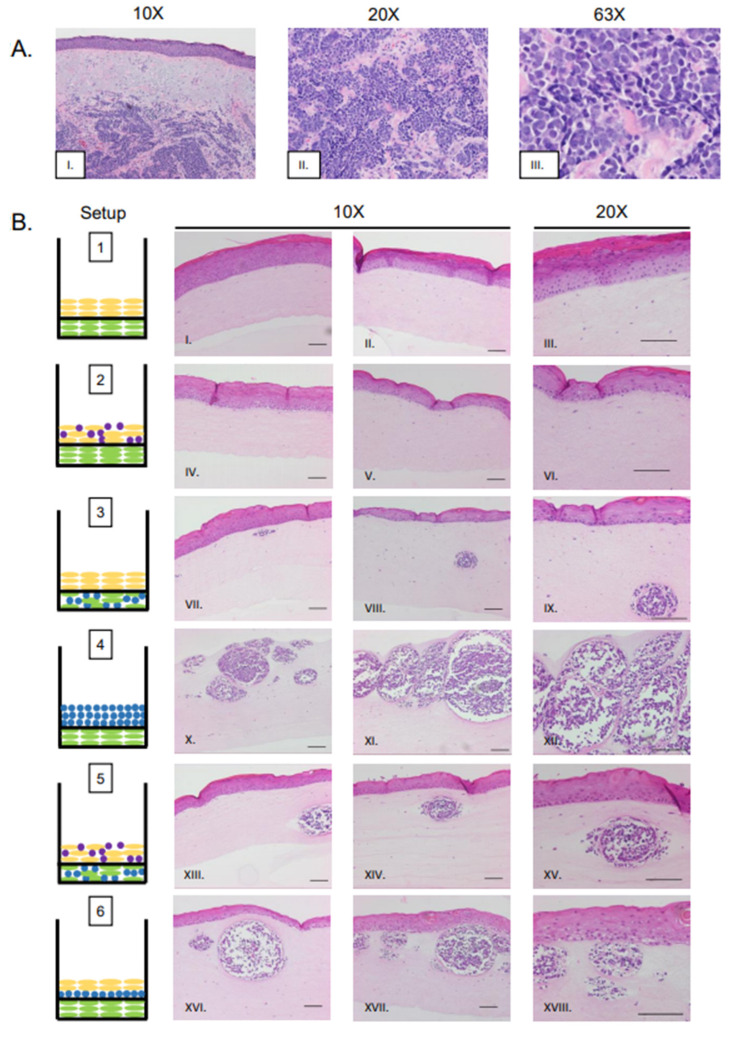
Histological analysis of MCPyV+ MCC-like lesions in organotypic raft cultures. (**A**) Representative images of H&E-stained tissue sections of human MCC tumors arising in the dermis of human skin (Panel I–III). (**B**) Representative H&E images of rafts generated using various culture conditions. Each raft setup is shown on the left and cell types are represented using the symbols outlined in Figure 1B. H&E-stained images are shown with 10× magnification in the first two columns (one each of both replicate rafts) and 20× magnification of histology in the rightmost column of one of those replicates (Panels I–XVIII). All scale bars = 100 µM.

In vitro studies show viral entry and infection in fibroblast cells, which are found within the dermal layer of the skin [8,33]. A majority of human MCC tumors develop within the dermal layer of human skin [53,54,55]. These findings suggest the dermis may be a preferential site for MCPyV-induced MCC biogenesis. Therefore, we designed rafts in which MCPyV+ MCC cells were embedded in collagen within the dermal equivalent (Figure 1B, Setups 3 and 5). Within the dermal layers, the formation of MCC-like lesions was observed (Figure 2B, Setup 3 Panel VII–IX, Setup 5 Panel XIII–XV), and these lesions were distributed intermittently throughout the dermal layer. These lesions contained small, nucleated cells with scant cytoplasm, a morphology that highly resembles that seen in human MCC (Figure 2A, Panel I–III) and is commonly observed in MCC histopathology [53,55]. These lesions were not present in control rafts generated using our standard protocol that lacked MCPyV+ MCC cells (Figure 2B, Setup 1 Panel I–III). 

Although our study identified several raft designs that produce these MCC-like lesions, one particular setup in which MCPyV+ MCC cells were embedded in collagen and added as an intermediate layer between the dermal equivalent and epithelial layer (Setup 6) appeared to most resemble MCCs as they are observed in humans. This setup (Figure 2B, Panel XVI–XVIII) also produced the highest frequency of lesions, and lesion size appeared greater in Setup 6 than in Setup 3 (Figure 2B, Panel VII–IX) or Setup 5 (Figure 2B, Panel XIII–XV). 

Merkel cells are present in the epidermis and arise from epithelial progenitor cells [56,57,58]. Up to 10% of MCC tumors show intra-epidermal lesions with no dermal involvement [59,60]. We questioned whether the MCC cell lines we are working with could create MCC-like lesions in the epidermis, or whether they migrate to the basal layer and/or are inclined to naturally invade into the dermis. We therefore included co-cultures where MCPyV+ MCC cells were mixed with NIKS keratinocytes to form the epidermal equivalent (Figure 1B, Setup 2). Histopathological analysis of these rafts show no MCC-like lesions in either the epidermal or dermal layers (Figure 2B, Panel IV–VI); rather, they resembled control rafts with only NIKS cells in the epithelial layer (Figure 1B, Setup 1; Figure 2B, Panel I–III). In those rafts where MCC cells were seeded into both the epidermal and dermal layers (Figure 1B, Setup 5; Figure 2B, Panel XIII–XV), it is unlikely that the MCC-like lesions that arose in the dermal equivalent originated from MCPyV+ MCC cells originally plated in the epithelial layer, as this characteristic was absent in rafts from Setup 2. 

We hypothesize that the absence of epidermal MCC-like lesions in Setup 2 might be due to the suspension nature of the MCC cell lines used in our studies. To determine the fate of these MCPyV+ MCC cells, the raft medium was removed before the lifting process on Day 11 (Figure 1A), and re-plated in a separate dish. The medium was monitored for the growth of cell clusters like those seen in MCPyV+ MCC monocultures. No clusters of MCC cells grew in the removed media (data not shown). This suggests to us that the MCC cells co-plated with the keratinocytes under Setup 2 neither survived within the raft itself nor in suspension under the culture conditions used. 

We also generated rafts where no NIKS cells were included and MCPyV+ MCC cells embedded in collagen were co-cultured on top of the dermal layer containing fibroblasts (Figure 1B; Setup 4). Large, oval MCC-like lesions still formed within the dermal equivalents (Figure 2B; Panel X–XII) even in the absence of keratinocytes. Within the lesions that develop, the general morphology of MCPyV+ MCC cells remain the same as those seen in Setup 3, 5, or 6. The MCPyV+ MCC cells within these lesions do not appear to migrate into the dermal equivalent based on our comparison of the distance between the bottom of the dermal equivalent and the epidermal layer in Setup 1 and the MCC-like lesions in Setup 4 (Supplemental Figure S2). We also assessed the necessity of fibroblasts in the development of these MCC-like lesions. We generated rafts without EF-1-F cells within the dermal equivalent or the MKL-1 collagen layer (Supplemental Figure S1A, Setups 7 and 8) and found that MCC-like lesions still formed within these rafts in the absence of fibroblasts (Supplemental Figure S1B, Setups 7 and 8 Panel I–III). Collectively, our results indicate that MCPyV+ MCC-like lesions can form within 3D in vitro organotypic rafts and their development in this model system is not dependent on the presence of keratinocytes or fibroblasts. 

### 3.3. Identification of MCPyV+ MCC Cells, Epithelial Cells, and Fibroblasts within Raft Structures

**Figure 3:**
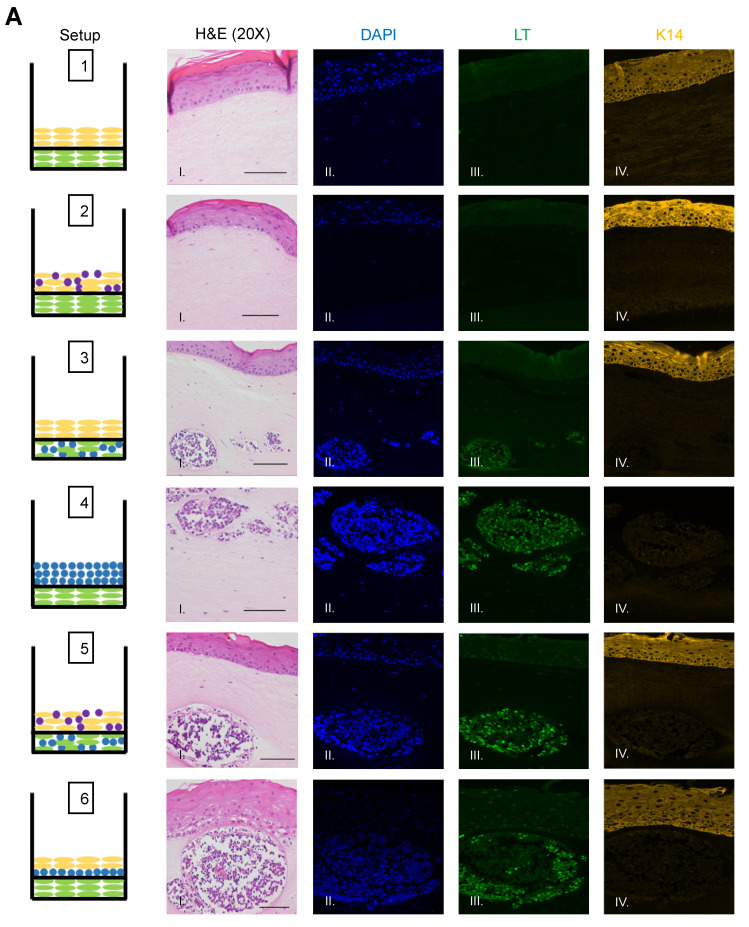

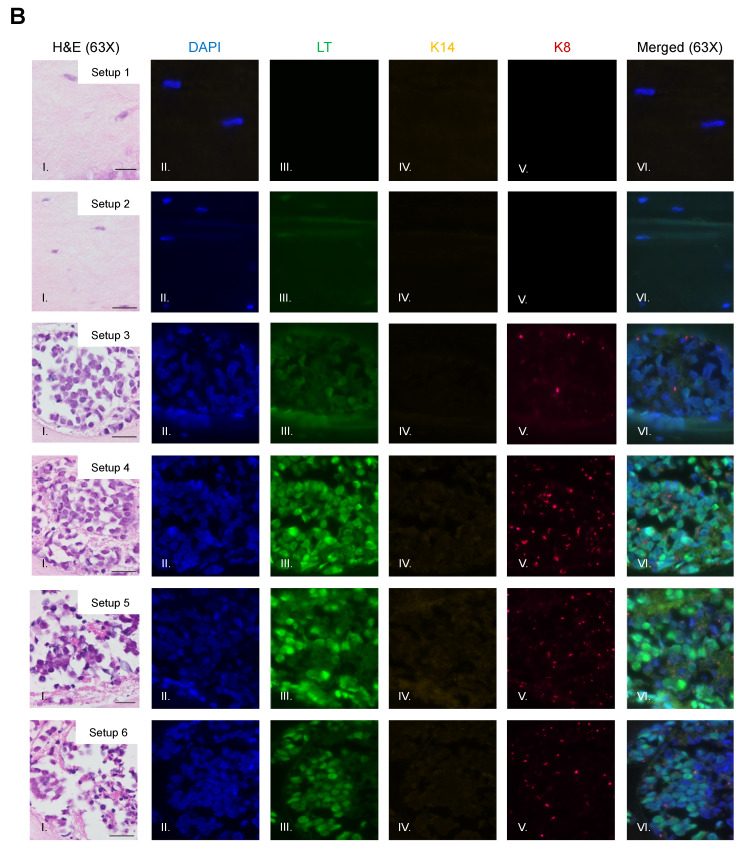
Identification of MCPyV+ MCC cells within raft structures. (**A**) Tissue sections from all raft setups were subjected to immunofluorescence analysis for LT antigen expression. DAPI counterstain was used to identify cell nuclei (blue, Column II), LT antigen was detected using the CM2B4 antibody (green, Column III), and K14 staining was used as an epithelial marker (yellow, Column IV). Corresponding H&E images are shown in Column I. All images are shown at 20× magnification. Scale bars = 100 µM. (**B**) High magnification (63×) of representative MCC-lesions that developed in rafts and IF biomarker analysis. Panel I—H&E, Panel II—DAPI (blue), Panel III—LT antigen (green), Panel IV—epithelial marker cytokeratin 14 (yellow), Panel V—cytokeratin 8 (pink). Merged IF images are shown in Panel VI. For rafts that do not develop these MCC lesions (Setup 1 and 2), representative images of the dermal layer are shown. Scale bars = 20 µM. Note in Setup 3 (panel III) LT staining was dimmer than in the MCC-like lesions that arose under other setups (4–6). We believe this is an artifact of the position of the MCC-like lesion under setup 3 toward the edge of the raft, where we often see a dimmer fluorescence signal.

To identify and confirm the cell types within each raft culture, we performed immunofluorescent analysis on the harvested tissue with several cell markers that distinguish MCPyV+ MCC cells, epithelial keratinocytes, and dermal fibroblasts, summarized in Table 1. To detect MCPyV+ MCC cells, we used the CM2B4 monoclonal antibody, which detects an epitope within exon 2 of MCPyV LT. We observed CM2B4 signals only within the MCC-like lesions that formed in the rafts (Figure 3A, Panel III). There was no detection of MCPyV+ MCC cells within the epithelial layer or the surrounding dermal layer, suggesting that individual MCPyV+ MCC cells or small clusters of cells did not establish residence outside of the MCC-like lesions. Keratin 8 (K8) is a well-defined marker for Merkel cells and has been used in studies on the development of Merkel cells [56,61,62] and for the identification of MCC in humans [63,64]. In addition to LT antigen, we stained for K8 to further verify that the MCC-like lesions retained shared Merkel cell and MCC characteristics. Within our rafts, we saw positive detection of K8-positive cells within the MCC-like dermal lesions but not in the epithelial layer or in surrounding dermal layer (Figure 3B). The pattern of the K8-positive signal was punctate and diffusely present throughout the dermal lesions (Figure 3B, Panel V), consistent with the pattern described in MCC histopathology [3]. The expression of K8 also co-localized with the expression of LT antigen (Figure 3B, Panel VI). Taken together with our immunofluorescence detection of LT antigen, our findings indicate that the MCC-like lesions that formed within the dermal equivalent of the rafts arise from MCPyV+ MCC cells. 

**Figure 4:**
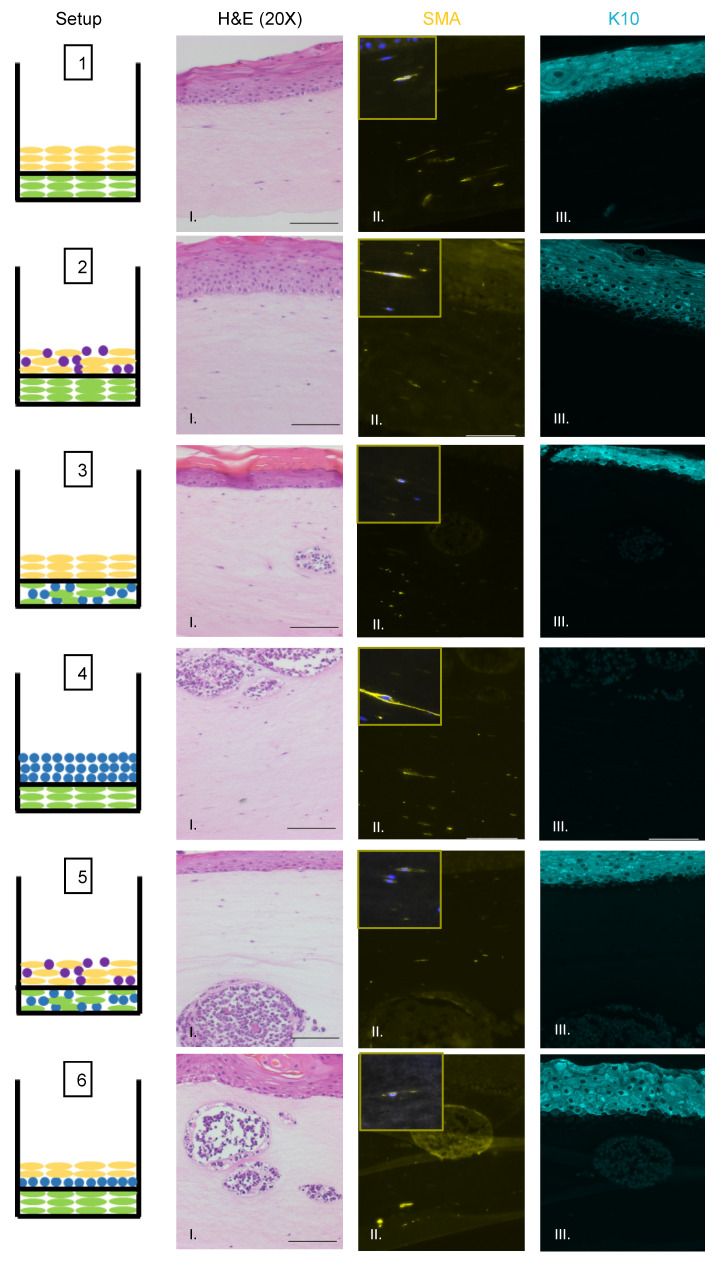
Identification of fibroblasts and differentiated keratinocytes within raft structures. Immunofluorescence analysis of tissue sections from all raft setups was performed using antibodies specific to the fibroblast marker α-smooth muscle actin (SMA) (yellow, Column II) and differentiated epithelial marker cytokeratin 10 (K10) (aqua, Column III). Corresponding H&E images are shown in Column I. Note for Setup 6 Panel II, the fluorescent surrounding the MCC lesion is likely background. Each raft setup is shown on the left and cell types are represented using the symbols outlined in Figure 1B. For SMA, 63× images of a positive fibroblast cell (with DAPI counterstain) are included as inset pictures in the top left corner. All scale bars = 100 µM.

We also observed no evidence that fibroblasts or epithelial cells were present within the MCC-like lesions. Immunofluorescence analysis for the epithelial markers cytokeratin 14 (K14) (Figure 3A), cytokeratin 10 (K10), and the fibroblast marker α-smooth muscle actin (SMA) was performed (Figure 4). K14 is expressed in the epithelial layers formed by keratinocytes whereas expression of K10 is restricted to the more differentiated suprabasal layers of the epithelium. During Merkel cell development, progenitor cells lose their epithelial phenotype as they progress through the Merkel cell differentiation pathway [65,66,67]. Both K14 (Figure 3A, Panel IV) and K10 (Figure 4, Panel III) showed positive signals within the epithelial equivalent of the rafts that contained NIKS (Figure 4, Setups 1,2,3,5,6), verifying the differentiation and stratification of the epithelial layer in our raft cultures. However, neither K14 nor K10 was detectable within the MCC-like lesions that formed within the dermal equivalent of any of the rafts (Figure 4, Setups 3–6). Likewise, there was no K14 or K10 signal detected at all in rafts where collagen-embedded MCPyV+ MCC cells were seeded on to the dermal equivalent and no NIKS keratinocytes were cultured (Figure 4, Setup 4). Overall, the absence of detection of keratinocyte markers is additional verification of Merkel cell traits within the MCC-like lesions arising in the dermal compartment of the rafts and distinguishes the lesions in our rafts from other cutaneous skin cancers such as squamous cell carcinoma or basal cell carcinoma. Strong SMA-positive cells were restricted to the individual fibroblasts scattered within the dermal equivalent (Figure 4, Panel II). While there was some background staining for SMA in the MCC-like lesions, we found no cells that stained strongly for SMA in those lesions, (Figure 4, Setups 3–6). The apparent SMA signal in the MCC-like lesions likely represents background since it was also observed when we omitted the primary antibody indicating the unlikelihood that fibroblasts became incorporated into such lesions arising in organotypic rafts. 

### 3.4. MCPyV+ MCC Cells Retain LT Function and Proliferative Capacity in Organotypic Raft Cultures

**Figure 5:**
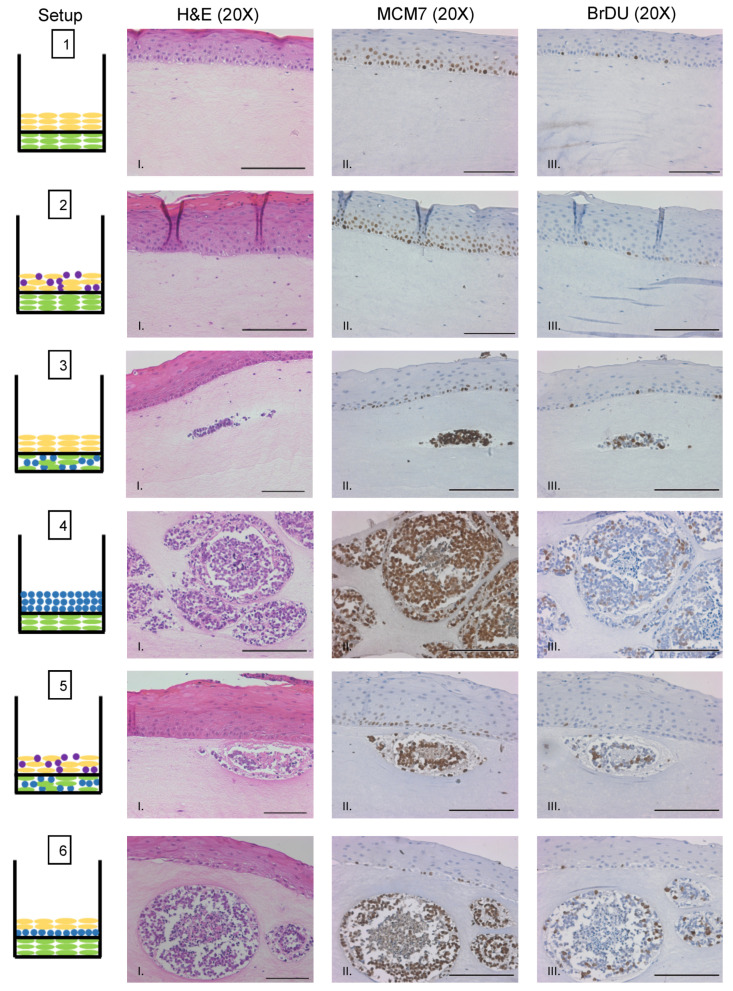
MCPyV+ MCC cells retain LT activity and proliferative capacity in organotypic raft cultures. Representative images showing immunohistochemical analysis for MCM7 (Panel II) and BrdU (Panel III) in raft setups are shown. Positive signal is indicated by brown nuclei staining. Counterstaining was done using hematoxylin (blue). Each raft setup is shown on the left and cell types are represented using the symbols outlined in Figure 1B. Corresponding H&E images are shown in Column I. All scale bars = 100 µM.

We sought to evaluate whether MCPyV+ MCC cells retain LT function and continue to proliferate when cultured in rafts. To assess LT antigen function, we performed immunohistochemistry on raft tissue sections for the nuclear mini-chromosome maintenance protein 7 (MCM7). *Mcm7* is an E2F-responsive gene whose expression can be used as a readout for the ability of the MCPyV LT antigen to bind and inactivate the cellular tumor suppressor Rb through its LxCxE motif, which is retained in MCC tumor-derived truncated LT antigens [16,17]. We previously found nuclear MCM7 staining is highly upregulated as a result of MCPyV T antigen expression in vivo [25]. Within the epithelial layer of rafts, nuclear MCM7 staining was restricted to the proliferative basal and parabasal cell layers (Figure 5, Setups 1–3, 5, and 6), which is the expected and normal expression pattern [68,69] and confirms the specificity of our antibody and the validity of the model. Within MCC-like lesions, we observed strong nuclear MCM7 staining in MCPyV+ MCC cells (Figure 5, Setups 3–6). These results indicate that the truncated LT antigen expressed in MCPyV+ MCC cells remains functional within the MCC-like lesions observed in 3D organotypic raft cultures. 

We also sought to verify that MCPyV+ MCC cells continue to proliferate when grown in raft cultures. Prior to harvesting rafts at the endpoint, we supplemented the culture media with bromodeoxyuridine (BrdU), a thymidine analog that is incorporated into DNA during cellular replication and can be used as a readout for cellular proliferation. We performed immunohistochemistry for BrdU on raft tissue sections (Figure 5). Similar to MCM7, BrdU exhibited the expected pattern of staining in the nuclei of highly proliferative basal cells of the epithelium. We also observed positive nuclear staining for BrdU within MCPyV+ MCC cells in the MCC-like lesions (Figure 5, Setups 3–6). We therefore conclude that MCPyV+ MCC cells not only retain LT function but also continue to proliferate when cultured in 3D organotypic rafts, both important and vital features necessary for any robust MCPyV+ MCC tissue culture model system. 

## 4. Conclusions

In this study, we describe the establishment of a three-dimensional in vitro model system that recapitulates the histopathology of MCPyV+ MCC in human skin. Through our histopathology and immunohistostaining analyses for MCPyV T antigen and Merkel cell biomarkers, as well as markers for keratinocytes and fibroblasts, we demonstrate that the MCC-like lesions that developed within the dermal equivalent are derived from proliferating MCPyV+ MCC cells, and not from epithelial cells or fibroblasts. The formation of these lesions was localized to the dermal layer of our rafts, similar to the majority of human MCC tumors [35,53,55]. Although our studies identified several raft setups that developed MCC-like lesions, we identified one (Setup 6), in which MCPyV+ MCC cells suspended in collagen between the epithelial and dermal equivalents, which best recapitulated the histopathology of human MCC, based on the high frequency and large size of the MCC-like lesions. Our 3D in vitro co-culture studies further illuminate that the development of MCC-like lesions from MCPyV+ MCC cells in organotypic rafts does not depend upon the presence of human keratinocytes. 

## 5. Discussion

In our studies, we observed that the formation of MCPyV+ MCC-like lesions occurs when cultured within the dermal equivalent of three-dimensional organotypic (raft) cultures. We showed that neither an epithelial cell component nor a fibroblast cell component was needed for MCC-like lesions to arise in the raft cultures; however, the presence of both together with the MCC cells placed within the dermal equivalent (i.e., Setup 6, Figure 2) gave rise to the largest and most abundant MCC-like lesions. Therefore, crosstalk between the different cell types may contribute qualitatively to the formation of the MCC-like lesions. MCPyV+ MCC did not appear to have any influence on the gross morphology or differentiation properties of the keratinocytes in the raft cultures as indicated by the immunohistochemical staining for the epithelial markers, K14 and K10, and the overall morphology of the epithelial layers arising under the various raft culture conditions. The same was true for fibroblasts. However, co-culture of these cells could be affecting certain molecular aspects of epithelial and fibroblast cell biology that are not yet appreciated. Therefore, the 3D tissue culture system presented could facilitate studies dissecting the involvement of signaling interactions between various skin cell types. Recent evidence suggests cellular interactions between keratinocytes and MCC cells in which T antigen is knocked down can induce neuronal morphological characteristics and activities, a phenotype that required direct cell–cell contact between keratinocytes and the MCC cells [28]. Other skin cell types could also be incorporated into our raft cultures. For instance, melanocytes and melanoma cells have been successfully incorporated into 3D rafts [70]. Prior studies into the carcinogenesis of melanoma has revealed the critical role of the Bcl-2 proteins and their capacity to confer apoptotic resistance to melanoma cancer cells (reviewed in [71]). This capability to study melanoma progression may be useful as prior evidence suggests that MCC cells express Bcl-2 at a higher level relative to normal Merkel cells in fetal and adult tissue [72,73]. Therefore, there may be some similarities between these two malignancies, which can be elucidated further in this tissue-relevant setting. Future studies using our 3D culture method can provide further insight into biological events that contribute to MCPyV-induced MCC tumorigenesis. Such studies could be pursued through genome-wide analyses, as well as genetic alteration and pharmacological manipulation of specific pathways and factors identified in such screens or by other studies.

Our 3D co-culture system also allows for investigations into the crosstalk and signaling interactions between the epidermal and dermal layers and whether they are necessary for MCPyV+ MCC development. For example, previous work in our lab elucidated a synergy between HPV16 E6 and E7 oncoproteins with stromal estrogen signaling that leads to the significant upregulation of inflammatory chemokine/cytokine expression associated with carcinogenesis [74]. Such interactions cannot be fully elucidated in in vitro monolayer culture or soft agar culture settings, and in vivo studies are limited due to the lack of a small animal MCPyV infection model. As such, rafts provide an attractive alternative for these studies given they fully recapitulate the three-dimensional stratification and differentiation of human skin. 

Raft cultures are extremely useful tools in the study of viral life cycles and oncogenesis [41,42,44,46,47,48,49,50,51,52,75]. We predict that the novel MCPyV+ MCC model system described here will also be useful. The rare incidence of MCC, despite the high prevalence of the virus, suggests that multiple factors/events are required for the initiation and development of MCPyV+ MCC. Not only does our model system recapitulate the histopathology of MCC, the activity of T antigens appears to be preserved as well, as seen by the functional inactivation of Rb within the lesions leading to high expression of nuclear MCM7. Therefore, this raft system would be ideal for studying co-factors that collaborate with the MCPyV T antigens to facilitate MCC biogenesis. Gain-of-function studies with transcription factor ATOH1 [20,26] and GLI1 [21], as well as loss-of-function studies with T antigens [28], have already provided insight into the potential mechanisms involved in this process. With our model, the biological consequences described in these studies, specifically cell morphology and behavior, can be explored further in a structurally and environmentally relevant model. 

A key area of research within the MCC field is in identifying the cell of origin. A significant amount of effort has been devoted to the identification of cell types that support viral entry, and within the skin, candidate cell types include epithelial cells and fibroblasts. To date, there are no current in vitro studies to show that these are the true cells of origin for MCC. Our raft system could be informative in investigating the cellular origins and viral integration events that lead to the development of MCC. Specifically, we could ask whether epithelial and/or fibroblasts transfected with MCPyV viral genomes and Merkel cell specification factors can give rise to MCC-like lesions in raft cultures (see also above) and whether the viral genome becomes integrated in these lesions. 

This recapitulation of the human skin microenvironment also allows for investigations into factors beyond cell–cell interactions that may affect the process of MCC tumorigenesis, such as collagen composition and stiffness. In breast cancer, extracellular matrix characteristics significantly alter tumor progression and metastasis, with increasing stiffness leading to more favorable conditions for vascularization, cell migration, and inflammation (reviewed in [76]). Furthermore, the microenvironment of the dermal layer can influence the transport of growth factors and cytokines, and this can be replicated in 3D tissue culture models (reviewed in [77,78]). The interplay between factors such as collagen density/stiffness and other microenvironment tissue dynamics and the formation, morphology, and overall biology of MCPyV+ MCC lesions is another facet of research that can be explored with our 3D tissue culture system.

As these rafts structurally mimic human MCC tumors, this culture system could be used to test therapeutic compounds. Paulson et al. predict a dramatic increase in the number of MCC cases by 2025 as a result of aging of the “Baby Boomer” generation [79]. MCC is notorious as a malignancy with high recurrence and metastatic rates, leading to overall poor prognosis of patients [80]. With a predicted increase in cases and the aggressive nature of the disease, the need for new therapies and ways to test them has become critical. Currently, MCC tumors are treated through radical forms of excision followed by radiotherapy and/or neo-adjuvant immunotherapy (reviewed in [54,81,82]). Traditionally, potential therapeutic strategies are most frequently tested in a small animal models before being expanded to clinical trials; the MCPyV+ MCC field currently lacks such an in vivo model. We therefore anticipate that our organotypic rafts will provide a much-needed platform to test cellular signal pathway inhibitors and antagonists. 

While having great promise, we acknowledge that this model has some limitations. Firstly, the cells included in our studies are limited to foreskin-derived keratinocytes and fibroblasts cells, and MCPyV+ MCC cells and exclude other potential cell types such as B cells and neuronal cells that have been identified as potential candidate cells types as the origin cell for MCC [10,28]. Additionally, the rafts reported here utilize only a single MCPyV+ MCC cell line. Additional studies to determine whether other classical MCPyV+ MCC cell lines can also be cultured in a similar fashion are necessary to further validate this co-culture system. To date, our experiments have only looked at MCPyV-positive MCC. We do not know whether our 3D culture system will allow us to recapitulate MCC-like lesions for MCPyV-negative MCCs, which account for 20% of MCC cases [83,84,85]. The latter cells tend to be more adherent in monolayer cultures, and so it will be interesting to see whether they grow as MCC-like lesions in the dermal equivalent. Additionally, our raft system does not incorporate other biological events that occur in the skin such as vascularization, immune cell infiltration, and the effects of UV irradiation. Transplanting these rafts on to immunosuppressed mouse strains may overcome, at least partially, such limitations. Lastly, we were not able to recapitulate the 10% of MCCs that arise within the epidermis. This may be a consequence of the particular MCC cell line we chose to use, MKL-1, which was derived from a metastatic MCC that normally grows in suspension, which may explain why it did not survive when co-cultured with keratinocytes in the epidermal equivalent. Further studies with additional MCC cell lines may address this limitation. We also did not observe any evidence for the MCC cell migration within the context of the rafts (Supplemental Figure S2). Therefore, as currently designed, we are unable to measure invasivity. Whether this can be successfully achieved may rely upon making variations in the composition of the extracellular matrix of the dermal equivalent. Regardless, the three-dimensional organotypic raft model of MCPyV+ MCC described in this report is poised to be utilized in a multitude of ways that will expand our knowledge of MCPyV+ MCC. 

## Figures and Tables

**Table 1:** Cell markers tested in immunofluorescent detection.

	MKL-1	NIKS	EF-1-F
LT antigen (CM2B4)	+	-	-
Cytokeratin 14 (K14)	−	+	−
Cytokeratin 10 (K10) *	−	+	−
Cytokeratin 8 (K8)	+	−	−
α-smooth muscle (SMA)	−	−	+

* K10 only detects differentiated epithelial layer from NIKS keratinocytes.

## Data Availability

The data presented in this study are available within this article.

## Supplementary Materials

The following are available online at https://www.mdpi.com/1999-4915/13/1/138/s1, Figure S1: Organotypic rafts without EF-1-F fibroblasts; Figure S2: Comparison of dermal equivalent thickness.

## References

[1] Feng H., Shuda M., Chang Y., Moore P.S. (2008). Clonal Integration of a Polyomavirus in Human Merkel Cell Carcinoma. Science.

[2] Toker C. (1972). Trabecular Carcinoma of the Skin. Arch. Dermatol..

[3] Becker J.C., Stang A., DeCaprio J.A., Cerroni L., Lebbe C., Veness M., Nghiem P. (2017). Merkel cell carcinoma. Nat. Rev. Dis. Prim..

[4] Cook L. (2016). Polyomaviruses. Microbiol. Spectr..

[5] DeCaprio J.A., Garcea R.L. (2013). A cornucopia of human polyomaviruses. Nat. Rev. Microbiol..

[6] Schowalter R.M., Pastrana D.V., Pumphrey K.A., Moyer A.L., Buck C.B. (2010). Merkel Cell Polyomavirus and Two Previously Unknown Polyomaviruses are Chronically Shed from Human Skin. Cell Host Microbe.

[7] Tolstov Y.L., Knauer A., Chen J.G., Kensler T.W., Kingsley L.A., Moore P.S., Chang Y. (2011). Asymptomatic primary Merkel cell polyomavirus infection among adults. Emerg. Infect. Dis..

[8] Liu W., Yang R., Payne A.S., Schowalter R.M., Spurgeon M.E., Lambert P.F., Xu X., Buck C.B., You J. (2016). Identifying the Target Cells and Mechanisms of Merkel Cell Polyomavirus Infection. Cell Host Microbe.

[9] Neumann F., Czech-Sioli M., Grundhoff A., Fischer N. (2015). In Vitro Replication Assay for Merkel Cell Polyomavirus (MCPyV). Curr. Protoc. Microbiol..

[10] Zur Hausen A., Rennspiess D., Winnepenninckx V., Speel E.J., Kurz A.K. (2013). Early B-cell differentiation in Merkel cell carcinomas: Clues to cellular ancestry. Cancer Res..

[11] Schowalter R.M., Reinhold W.C., Buck C.B. (2012). Entry tropism of BK and Merkel cell polyomaviruses in cell culture. PLoS ONE.

[12] Neumann F., Borchert S., Schmidt C., Reimer R., Hohenberg H., Fischer N., Grundhoff A. (2011). Replication, Gene Expression and Particle Production by a Consensus Merkel Cell Polyomavirus (MCPyV) Genome. PLoS ONE.

[13] Kwun H.J., Chang Y., Moore P.S. (2017). Protein-mediated viral latency is a novel mechanism for Merkel cell polyomavirus persistence. Proc. Natl. Acad. Sci. USA.

[14] Moens U., Rasheed K., Abdulsalam I., Sveinbjørnsson B. (2015). The Role of Merkel Cell Polyomavirus and Other Human Polyomaviruses in Emerging Hallmarks of Cancer. Viruses.

[15] Schrama D., Sarosi E.M., Adam C., Ritter C., Kaemmerer U., Klopocki E., König E.M., Utikal J., Becker J.C., Houben R. (2019). Characterization of six Merkel cell polyomavirus-positive Merkel cell carcinoma cell lines: Integration pattern suggest that large T antigen truncating events occur before or during integration. Int. J. Cancer.

[16] Shuda M., Feng H., Kwun H.J., Rosen S.T., Gjoerup O., Moore P.S., Chang Y. (2008). T antigen mutations are a human tumor-specific signature for Merkel cell polyomavirus. Proc. Natl. Acad. Sci. USA.

[17] Hesbacher S., Pfitzer L., Wiedorfer K., Angermeyer S., Borst A., Haferkamp S., Scholz C.J., Wobser M., Schrama D., Houben R. (2016). RB1 is the crucial target of the Merkel cell polyomavirus Large T antigen in Merkel cell carcinoma cells. Oncotarget.

[18] Pilon A.A., Desjardins P., Hassell J.A., Mes-Masson A.M. (1996). Functional implications of mutations within polyomavirus large T antigen Rb-binding domain: Effects on pRb and p107 binding in vitro and immortalization activity in vivo. J. Virol..

[19] Cheng J., Rozenblatt-Rosen O., Paulson K.G., Nghiem P., DeCaprio J.A. (2013). Merkel cell polyomavirus large T antigen has growth-promoting and inhibitory activities. J. Virol..

[20] Fan K., Gravemeyer J., Ritter C., Rasheed K., Gambichler T., Moens U., Shuda M., Schrama D., Becker J.C. (2020). MCPyV Large T Antigen-Induced Atonal Homolog 1 is a Lineage-Dependency Oncogene in Merkel Cell Carcinoma. J. Investig. Dermatol..

[21] Kervarrec T., Samimi M., Hesbacher S., Berthon P., Wobser M., Sallot A., Sarma B., Schweinitzer S., Gandon T., Destrieux C. (2020). Merkel Cell Polyomavirus T Antigens Induce Merkel Cell-Like Differentiation in GLI1-Expressing Epithelial Cells. Cancers.

[22] Kwun H.J., Shuda M., Feng H., Camacho C.J., Moore P.S., Chang Y. (2013). Merkel Cell Polyomavirus Small T Antigen Controls Viral Replication and Oncoprotein Expression by Targeting the Cellular Ubiquitin Ligase SCFFbw7. Cell Host Microbe.

[23] Shuda M., Guastafierro A., Geng X., Shuda Y., Ostrowski S.M., Lukianov S., Jenkins F.J., Honda K., Maricich S.M., Moore P.S. (2015). Merkel Cell Polyomavirus Small T Antigen Induces Cancer and Embryonic Merkel Cell Proliferation in a Transgenic Mouse Model. PLoS ONE.

[24] Shuda M., Kwun H.J., Feng H., Chang Y., Moore P.S. (2011). Human Merkel cell polyomavirus small T antigen is an oncoprotein targeting the 4E-BP1 translation regulator. J. Clin. Investig..

[25] Spurgeon M.E., Cheng J., Bronson R.T., Lambert P.F., DeCaprio J.A. (2015). Tumorigenic activity of merkel cell polyomavirus T antigens expressed in the stratified epithelium of mice. Cancer Res..

[26] Verhaegen M.E., Mangelberger D., Harms P.W., Eberl M., Wilbert D.M., Meireles J., Bichakjian C.K., Saunders T.L., Wong S.Y., Dlugosz A.A. (2017). Merkel Cell Polyomavirus Small T Antigen Initiates Merkel Cell Carcinoma-like Tumor Development in Mice. Cancer Res..

[27] Houben R., Shuda M., Weinkam R., Schrama D., Feng H., Chang Y., Moore P.S., Becker J.C. (2010). Merkel Cell Polyomavirus-Infected Merkel Cell Carcinoma Cells Require Expression of Viral T Antigens. J. Virol..

[28] Harold A., Amako Y., Hachisuka J., Bai Y., Li M.Y., Kubat L., Gravemeyer J., Franks J., Gibbs J.R., Park H.J. (2019). Conversion of Sox2-dependent Merkel cell carcinoma to a differentiated neuron-like phenotype by T antigen inhibition. Proc. Natl. Acad. Sci. USA.

[29] Guastafierro A., Feng H., Thant M., Kirkwood J.M., Chang Y., Moore P.S., Shuda M. (2013). Characterization of an early passage Merkel cell polyomavirus-positive Merkel cell carcinoma cell line, MS-1, and its growth in NOD scid gamma mice. J. Virol. Methods.

[30] Martin E.M., Gould V.E., Hoog A., Rosen S.T., Radosevich J.A., Deftos L.J. (1991). Parathyroid hormone-related protein, chromogranin A, and calcitonin gene products in the neuroendocrine skin carcinoma cell lines MKL1 and MKL2. Bone Miner..

[31] Rosen S.T., Gould V.E., Salwen H.R., Herst C.V., Le Beau M.M., Lee I., Bauer K., Marder R.J., Andersen R., Kies M.S. (1987). Establishment and characterization of a neuroendocrine skin carcinoma cell line. Lab. Investig..

[32] Velásquez C., Amako Y., Harold A., Toptan T., Chang Y., Shuda M. (2018). Characterization of a Merkel Cell Polyomavirus-Positive Merkel Cell Carcinoma Cell Line CVG-1. Front. Microbiol..

[33] Becker M., Dominguez M., Greune L., Soria-Martinez L., Pfleiderer M.M., Schowalter R., Buck C.B., Blaum B.S., Schmidt M.A., Schelhaas M. (2019). Infectious Entry of Merkel Cell Polyomavirus. J. Virol..

[34] Koljonen V. (2006). Merkel cell carcinoma. World J. Surg. Oncol..

[35] Swann M.H., Yoon J. (2007). Merkel Cell Carcinoma. Semin. Oncol..

[36] Asselineau D., Prunieras M. (1984). Reconstruction of ‘simplified’ skin: Control of fabrication. Br. J. Dermatol..

[37] Aasen T., Hodgins M.B., Edward M., Graham S.V. (2003). The relationship between connexins, gap junctions, tissue architecture and tumour invasion, as studied in a novel in vitro model of HPV-16-associated cervical cancer progression. Oncogene.

[38] Allen-Hoffmann B.L., Schlosser S.J., Ivarie C.A.R., Meisner L.F., O’Connor S.L., Sattler C.A. (2000). Normal Growth and Differentiation in a Spontaneously Immortalized Near-Diploid Human Keratinocyte Cell Line, NIKS. J. Investig. Dermatol..

[39] Flores E.R., Allen-Hoffmann B.L., Lee D., Sattler C.A., Lambert P.F. (1999). Establishment of the human papillomavirus type 16 (HPV-16) life cycle in an immortalized human foreskin keratinocyte cell line. Virology.

[40] Lambert P.F., Ozbun M.A., Collins A., Holmgren S., Lee D., Nakahara T. (2005). Using an immortalized cell line to study the HPV life cycle in organotypic “raft” cultures. Methods Mol. Med..

[41] Lee D., Norby K., Hayes M., Chiu Y.F., Sugden B., Lambert P.F. (2016). Using Organotypic Epithelial Tissue Culture to Study the Human Papillomavirus Life Cycle. Curr. Protoc. Microbiol..

[42] Meyers C. (1996). Organotypic (raft) epithelial tissue culture system for the differentiation-dependent replication of papillomavirus. Methods Cell Sci..

[43] Meyers C., Frattini M.G., Hudson J.B., Laimins L.A. (1992). Biosynthesis of human papillomavirus from a continuous cell line upon epithelial differentiation. Science.

[44] Nakahara T., Peh W.L., Doorbar J., Lee D., Lambert P.F. (2005). Human Papillomavirus Type 16 E1^E4 Contributes to Multiple Facets of the Papillomavirus Life Cycle. J. Virol..

[45] Hukkanen V., Mikola H., Nykänen M., Syrjänen S. (1999). Herpes simplex virus type 1 infection has two separate modes of spread in three-dimensional keratinocyte culture. J. Gen. Virol..

[46] Visalli R.J., Courtney R.J., Meyers C. (1997). Infection and Replication of Herpes Simplex Virus Type 1 in an Organotypic Epithelial Culture System. Virology.

[47] Meyers C., Mane M., Kokorina N., Alam S., Hermonat P.L. (2000). Ubiquitous Human Adeno-Associated Virus Type 2 Autonomously Replicates in Differentiating Keratinocytes of a Normal Skin Model. Virology.

[48] Andrei G., van den Oord J., Fiten P., Opdenakker G., De Wolf-Peeters C., De Clercq E., Snoeck R. (2005). Organotypic Epithelial Raft Cultures as a Model for Evaluating Compounds against Alphaherpesviruses. Antimicrob. Agents Chemother..

[49] Makielski K.R., Lee D., Lorenz L.D., Nawandar D.M., Chiu Y.F., Kenney S.C., Lambert P.F. (2016). Human papillomavirus promotes Epstein-Barr virus maintenance and lytic reactivation in immortalized oral keratinocytes. Virology.

[50] Nawandar D.M., Ohashi M., Djavadian R., Barlow E., Makielski K., Ali A., Lee D., Lambert P.F., Johannsen E., Kenney S.C. (2017). Differentiation-Dependent LMP1 Expression Is Required for Efficient Lytic Epstein-Barr Virus Reactivation in Epithelial Cells. J. Virol..

[51] Nawandar D.M., Wang A., Makielski K., Lee D., Ma S., Barlow E., Reusch J., Jiang R., Wille C.K., Greenspan D. (2015). Differentiation-Dependent KLF4 Expression Promotes Lytic Epstein-Barr Virus Infection in Epithelial Cells. PLoS Pathog..

[52] Temple R.M., Zhu J., Budgeon L., Christensen N.D., Meyers C., Sample C.E. (2014). Efficient replication of Epstein–Barr virus in stratified epithelium in vitro. Proc. Natl. Acad. Sci. USA.

[53] Harms P.W. (2017). Update on Merkel Cell Carcinoma. Clin. Lab. Med..

[54] Harms P.W., Harms K.L., Moore P.S., De Caprio J.A., Nghiem P., Wong M.K.K., Brownell I., International Workshop on Merkel Cell Carcinoma Research Working Group (2018). The biology and treatment of Merkel cell carcinoma: Current understanding and research priorities. Nat. Rev. Clin. Oncol..

[55] Smith P.D., Patterson J.W. (2001). Merkel cell carcinoma (neuroendocrine carcinoma of the skin). Am. J. Clin. Pathol..

[56] Maricich S.M., Wellnitz S.A., Nelson A.M., Lesniak D.R., Gerling G.J., Lumpkin E.A., Zoghbi H.Y. (2009). Merkel Cells Are Essential for Light-Touch Responses. Science.

[57] Moll I., Zieger W., Schmelz M. (1996). Proliferative merkel cells were not detected in human skin. Arch. Dermatol. Res..

[58] Morrison K.M., Miesegaes G.R., Lumpkin E.A., Maricich S.M. (2009). Mammalian Merkel cells are descended from the epidermal lineage. Dev. Biol..

[59] Brown H.A., Sawyer D.M., Woo T. (2000). Intraepidermal Merkel cell carcinoma with no dermal involvement. Am. J. Dermatopathol..

[60] Jour G.A.-O., Aung P.P., Rozas-Muñoz E., Curry J.L., Prieto V., Ivan D. (2017). Intraepidermal Merkel cell carcinoma: A case series of a rare entity with clinical follow up. J. Cutan. Pathol..

[61] Ostrowski S.M., Wright M.C., Bolock A.M., Geng X., Maricich S.M. (2015). Ectopic Atoh1 expression drives Merkel cell production in embryonic, postnatal and adult mouse epidermis. Development.

[62] Wright M.C., Reed-Geaghan E.G., Bolock A.M., Fujiyama T., Hoshino M., Maricich S.M. (2015). Unipotent, Atoh1+ progenitors maintain the Merkel cell population in embryonic and adult mice. J. Cell Biol..

[63] Moll R., Osborn M., Hartschuh W., Moll I., Mahrle G., Weber K. (1986). Variability of expression and arrangement of cytokeratin and neurofilaments in cutaneous neuroendocrine carcinomas (Merkel cell tumors): Immunocytochemical and biochemical analysis of twelve cases. Ultrastruct. Pathol..

[64] Schmidt U., Müller U., Metz K.A., Leder L.D. (1998). Cytokeratin and neurofilament protein staining in Merkel cell carcinoma of the small cell type and small cell carcinoma of the lung. Am. J. Dermatopathol..

[65] Nguyen M.B., Cohen I., Kumar V., Xu Z., Bar C., Dauber-Decker K.L., Tsai P.C., Marangoni P., Klein O.D., Hsu Y.C. (2018). FGF signalling controls the specification of hair placode-derived SOX9 positive progenitors to Merkel cells. Nat. Commun..

[66] Perdigoto C.N., Bardot E.S., Valdes V.J., Santoriello F.J., Ezhkova E. (2014). Embryonic maturation of epidermal Merkel cells is controlled by a redundant transcription factor network. Development.

[67] Xiao Y., Thoresen D.T., Miao L., Williams J.S., Wang C., Atit R.P., Wong S.Y., Brownell I. (2016). A Cascade of Wnt, Eda, and Shh Signaling Is Essential for Touch Dome Merkel Cell Development. PLoS Genet..

[68] Hiraiwa A., Fujita M., Nagasaka T., Adachi A., Ohashi M., Ishibashi M. (1997). Immunolocalization of hCDC47 protein in normal and neoplastic human tissues and its relation to growth. Int. J. Cancer.

[69] Suzuki S., Adachi A., Hiraiwa A., Ohashi M., Ishibashi M., Kiyono T. (1998). Cloning and characterization of human MCM7 promoter. Gene.

[70] Meier F., Nesbit M., Hsu M.-Y., Martin B., Van Belle P., Elder D.E., Schaumburg-Lever G., Garbe C., Walz T.M., Donatien P. (2000). Human Melanoma Progression in Skin Reconstructs: Biological Significance of bFGF. Am. J. Pathol..

[71] Eberle J., Hossini A.M. (2008). Expression and function of bcl-2 proteins in melanoma. Curr. Genom..

[72] Moll I., Gillardon F., Waltering S., Schmelz M., Moll R. (1996). Differences of bcl-2 protein expression between Merkel cells and Merkel cell carcinomas. J. Cutan. Pathol..

[73] Plettenberg A., Pammer J., Tschachler E. (1996). Merkel cells and Merkel cell carcinoma express the BCL-2 proto-oncogene. Exp. Dermatol..

[74] Spurgeon M.E., den Boon J.A., Horswill M., Barthakur S., Forouzan O., Rader J.S., Beebe D.J., Roopra A., Ahlquist P., Lambert P.F. (2017). Human papillomavirus oncogenes reprogram the cervical cancer microenvironment independently of and synergistically with estrogen. Proc. Natl. Acad. Sci. USA.

[75] Flores E.R., Allen-Hoffmann B.L., Lee D., Lambert P.F. (2000). The Human Papillomavirus Type 16 E7 Oncogene Is Required for the Productive Stage of the Viral Life Cycle. J. Virol..

[76] Kaushik S., Pickup M.W., Weaver V.M. (2016). From transformation to metastasis: Deconstructing the extracellular matrix in breast cancer. Cancer Metastasis Rev..

[77] Infanger D.W., Lynch M.E., Fischbach C. (2013). Engineered Culture Models for Studies of Tumor-Microenvironment Interactions. Ann. Rev. Biomed. Eng..

[78] Kalli M., Stylianopoulos T. (2018). Defining the Role of Solid Stress and Matrix Stiffness in Cancer Cell Proliferation and Metastasis. Front. Oncol..

[79] Paulson K.G., Park S.Y., Vandeven N.A., Lachance K., Thomas H., Chapuis A.G., Harms K.L., Thompson J.A., Bhatia S., Stang A. (2018). Merkel cell carcinoma: Current US incidence and projected increases based on changing demographics. J. Am. Acad. Dermatol..

[80] Liang E., Brower J.V., Rice S.R., Buehler D.G., Saha S., Kimple R.J. (2015). Merkel Cell Carcinoma Analysis of Outcomes: A 30-Year Experience. PLoS ONE.

[81] Tello T.L., Coggshall K., Yom S.S., Yu S.S. (2018). Merkel cell carcinoma: An update and review: Current and future therapy. J. Am. Acad. Dermatol..

[82] Wang T.S., Byrne P.J., Jacobs L.K., Taube J.M. (2011). Merkel cell carcinoma: Update and review. Semin. Cutan. Med. Surg..

[83] Harms P.W., Vats P., Verhaegen M.E., Robinson D.R., Wu Y.-M., Dhanasekaran S.M., Palanisamy N., Siddiqui J., Cao X., Su F. (2015). The Distinctive Mutational Spectra of Polyomavirus-Negative Merkel Cell Carcinoma. Cancer Res..

[84] Starrett G.J., Thakuria M., Chen T., Marcelus C., Cheng J., Nomburg J., Thorner A.R., Slevin M.K., Powers W., Burns R.T. (2020). Clinical and molecular characterization of virus-positive and virus-negative Merkel cell carcinoma. Genome Med..

[85] Wong S.Q., Waldeck K., Vergara I.A., Schröder J., Madore J., Wilmott J.S., Colebatch A.J., De Paoli-Iseppi R., Li J., Lupat R. (2015). UV-Associated Mutations Underlie the Etiology of MCV-Negative Merkel Cell Carcinomas. Cancer Res..

