# Supplementary material for: A Novel In Vitro Culture Model System to Study Merkel Cell Polyomavirus–Associated MCC Using Three-Dimensional Organotypic Raft Equivalents of Human Skin
Source: Viruses. 2021 Jan 19;13(1):138. doi: 10.3390/v13010138 (PMC7835998; doi:10.3390/v13010138)
Supplement: Supplementary file 1 [file viruses-13-00138-s001.pdf]

Supp 1A.

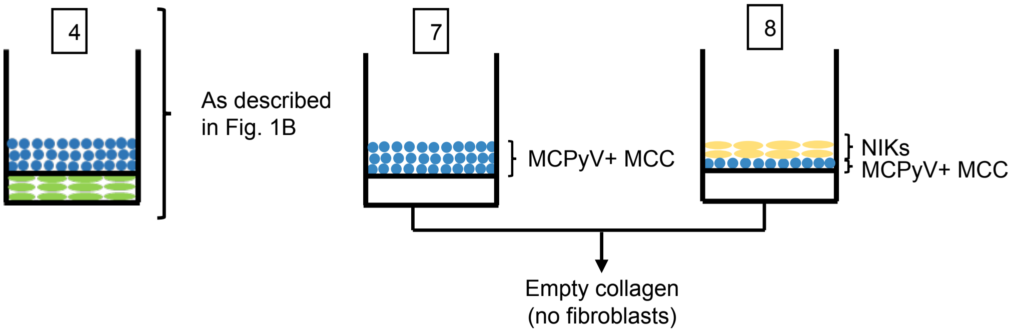

Supp 1B.

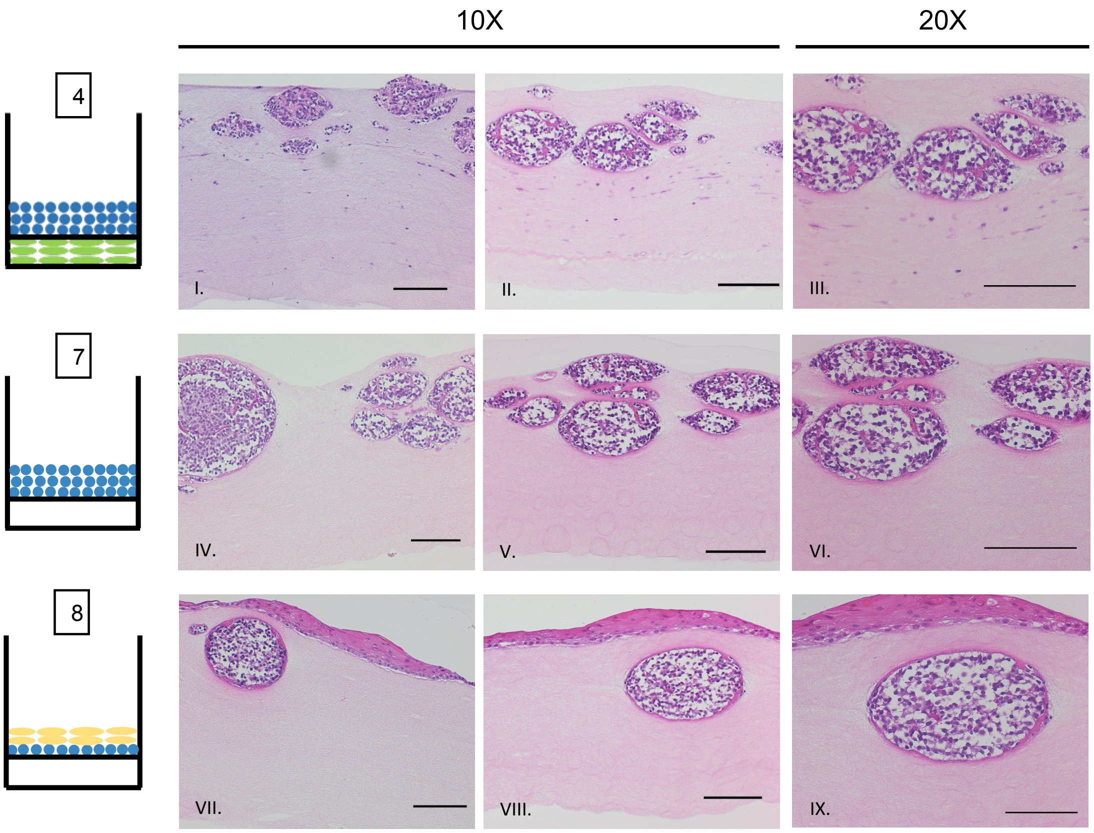

Supplemental Figure 1: Organotypic rafts without EF-1-F fibroblasts. A) Experimental organization/setups of rafts generated without EF-1-F fibroblasts. Cell types included in the various raft setups include NIKs keratinocytes (yellow), EF-1-F fibroblasts (green), and MCPyV+ MCC cells in collagen (blue). B) Representative H&E images of rafts generated from Setup 4, 7 and 8. Each raft setup is shown on the left and cell types are represented using the symbols outlined in Main Figure Figure 1B. H&E-stained images are shown with 10X magnification in first two columns (representative images of two replicate rafts per each condition - panels i,ii, iv, v, vii, viii) and 20X magnification of histology in the rightmost column of one of those replicates (panels ii, vi, ix). All scale bars = 100 μM.

Supp 2.

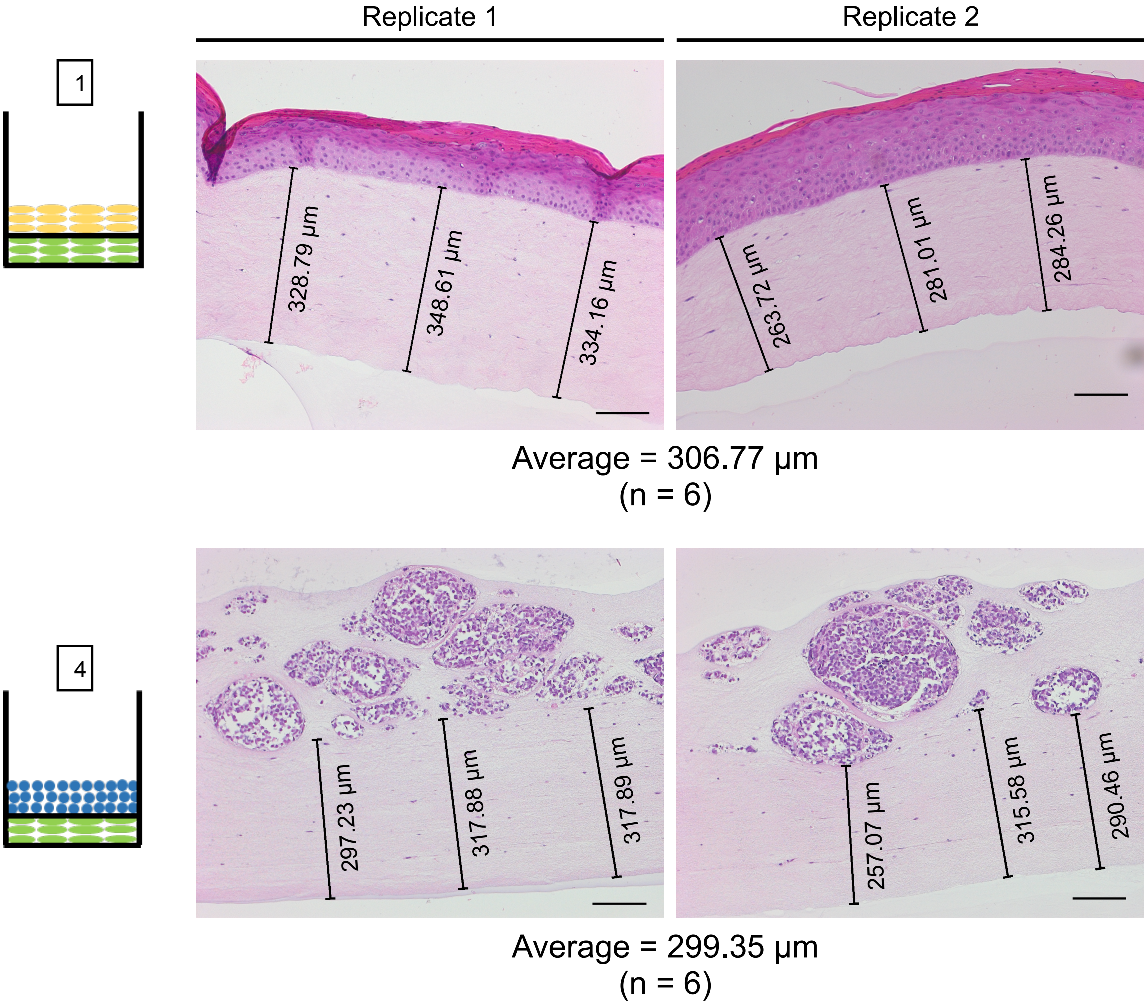

Supplemental Figure 2: Comparison of dermal equivalent thickness Measured dermal equivalents comparing rafts from Setup 1 (top) and Setup 4 (bottom). Measurements were taken from each replicate. All scale bars = 100 µM.
